# Rare Case of Gout Leading to Septic Arthritis, Osteomyelitis, and Septic Shock in an Elderly Patient

**DOI:** 10.7759/cureus.48836

**Published:** 2023-11-15

**Authors:** Yousra Gheit, Ibrahim S Gheit, Joseph Ierulli, Ines Mbaga

**Affiliations:** 1 Internal Medicine, Florida Atlantic University Charles E. Schmidt College of Medicine, Boca Raton, USA; 2 Neurosciences, Florida Atlantic University, Jupiter, USA; 3 Infectious Disease, Florida Atlantic University Charles E. Schmidt College of Medicine, Boca Raton, USA

**Keywords:** antibiotic administration in the management of severe sepsis, gouty arthritis, hospice and palliative care, gout flare, tophaceous gout

## Abstract

Gout is a common, chronic inflammatory arthritis that oftentimes accompanies an initial acute and painful attack characterized by intense pain and swelling. Although it may present in different sites such as the ankles, wrists, knees, elbows, and fingers, the lower extremities are the most common site of involvement. The pathophysiology of gout is complex, but typically, the deposition of monosodium urate crystals within the joint space and the subsequent acute inflammatory response play an important role. Following an acute attack, chronic gout can present with tophi or nests of monosodium urate crystals surrounded by macrophages and multinucleated giant cells that trigger granulomatous inflammation. Progressively, chronic gout can lead to several other complications including joint destruction, gout nephropathy, spinal compression, and secondary infections. In this case report, we present an elderly female patient with chronic gout and multiple tophi formations in all digits of both of her hands. The tophi led to an ulceration and secondary septic arthritis and osteomyelitis of the right second digit. By the time the patient presented and was admitted to the hospital, she was in septic shock. We will review the pathogenesis of gout and other cases of concomitant septic arthritis and gout, as well as medical management and necessary surgical intervention as a means of treatment.

## Introduction

Gout is one of the most common causes of chronic inflammatory arthritis in the United States. In general, gout results from the precipitation and accumulation of monosodium urate (MSU) monohydrate crystals within a joint space leading to an acute inflammatory response involving neutrophils. The causes of deposition of MSU are complex and involve a combination of several factors. These factors may encompass genetic predisposition, medical comorbidities, and dietary factors such as consuming high amounts of alcohol, certain seafoods, and red meats [1]. Specifically, several genes have been identified as being associated with gout and the heritability of hyperuricemia. In fact, approximately 40-50% of patients with gout have a family history of the disease. 

Hyperuricemia is an important pathophysiological factor and is a crucial factor for developing gout. In certain cases, hyperuricemia may be a result of the genetic absence of uricase or increased reabsorption of filtered uric acid. The limited solubility of MSU and urate in body fluids eventually leads to its deposition in joints. Particularly, tophi are nests of MSU crystals surrounded by macrophages and multinucleated giant cells that trigger granulomatous inflammation. Eventually, this leads to erosions of the cartilage and bone in the joint space [1]. 

Gout affects about 3.9% of adults in the United States or 9.2 million people. The clinical presentation of gout typically includes an acute gout attack, marked by the sudden emergence of intense pain and swelling, and it oftentimes occurs in the lower extremities. Thereafter, chronic, advanced gout may result in complications such as tophi, joint deformity, osteoarthritis, and bone loss [1]. Arthritis is the result of joint damage caused by the accumulation of uric acid crystals that is left untreated. Occasionally, this can lead to secondary septic arthritis, either by local infection due to skin breakdown or through hematogenous spread from another source. Gouty arthritis presents a challenge in diagnosis when it becomes infected, especially in patients with open or local ulcerations. This is because it is oftentimes difficult to distinguish between gout presenting with a superficial infection and gout with secondary septic arthritis/osteomyelitis, as both scenarios present radiologically as destruction of the joint. 

Typically, aspiration of synovial fluid for septic arthritis will yield a higher white blood cell (WBC) count (over 50,000) than in gout alone. It usually also accompanies a positive Gram stain and cultures of bacteria. We present a case of chronic gout with multiple tophi formations in all digits of the hands that led to ulcerations and secondary septic arthritis and osteomyelitis of the right second digit. This unfortunately progressed to a severe presentation of sepsis and septic shock in an elderly patient with otherwise limited past medical history.

## Case presentation

The patient is a 100-year-old female with a past medical history significant for hypertension, a pacemaker placement approximately 30 years ago per caregiver, and a chronic, poorly controlled history of gout with tophi, who presented to the emergency department and was admitted for altered mental status per her caregiver and son. Although the patient was not interactive at bedside, the son stated that she complained of neck pain, headache, nausea, and vomiting for four days prior to arrival at the hospital. She progressively became incoherent in speech and altered in mentation. Additionally, the caregiver noticed that the patient had a long-standing history of tophi to all digits of her bilateral hands but was complaining of right second digit pain and an ulceration with associated white fluid drainage from the tophus since the onset of her systemic symptoms.

**Figure 1:**
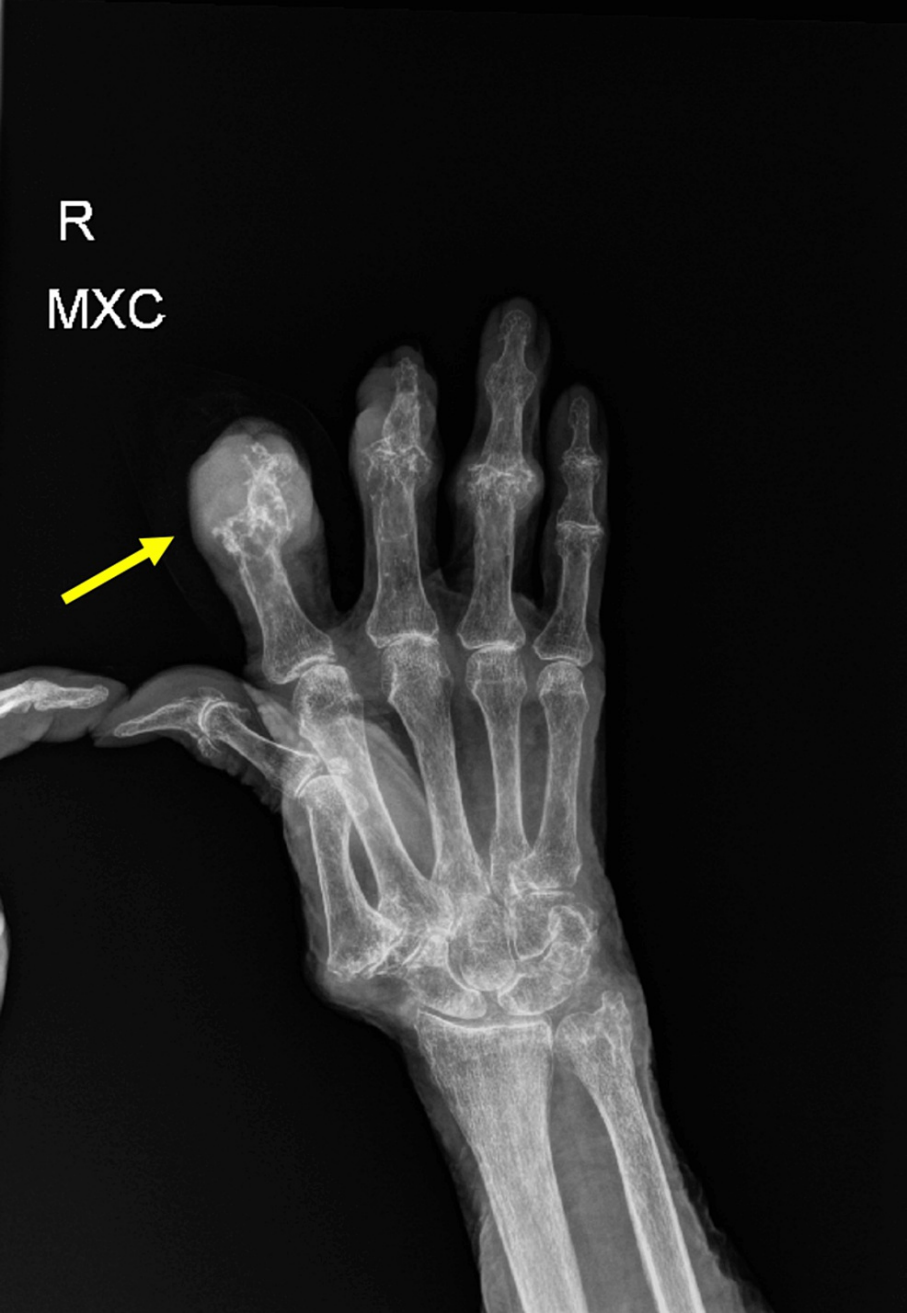
X-ray of the right second digit depicting marked soft tissue thickening with underlying bony destruction at the second digit.

**Figure 2:**
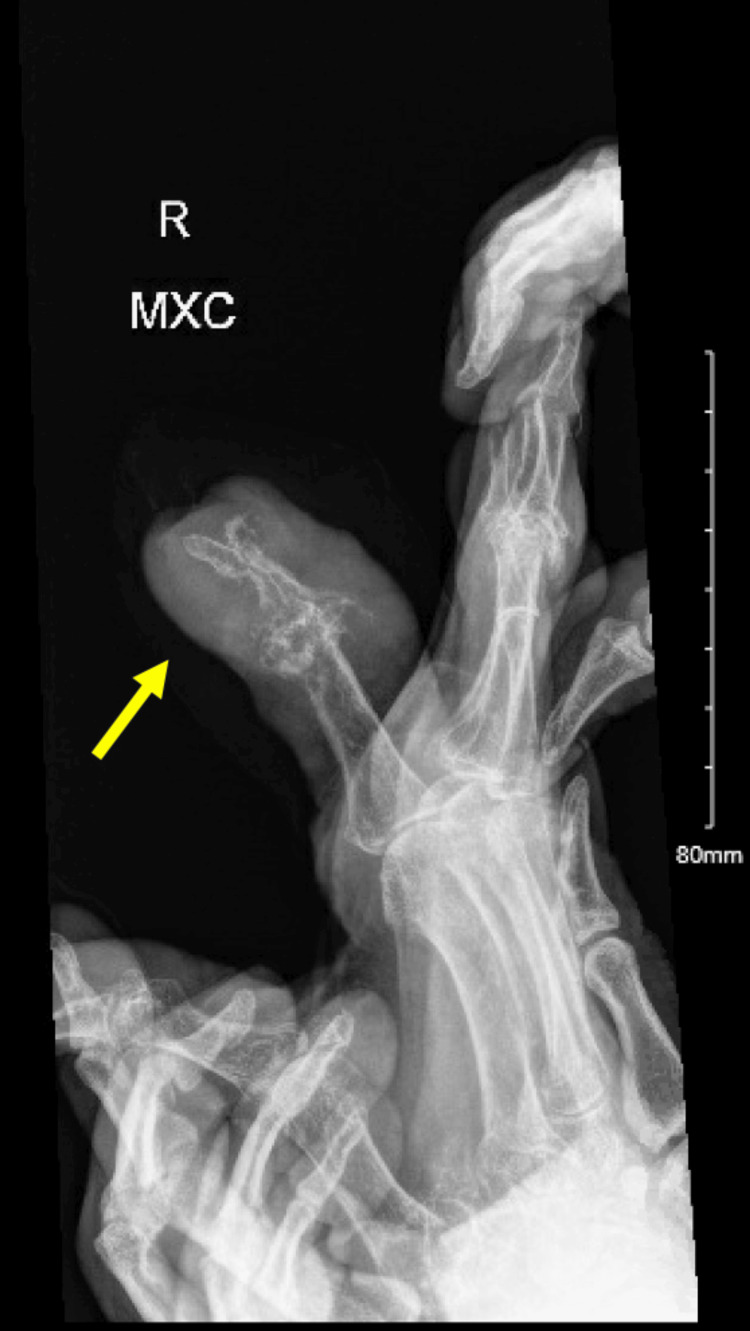
Lateral view X-ray depicting marked soft tissue thickening with underlying bony destruction at the second digit.

On presentation, the patient's vital signs were remarkable with a body temperature of 35.9 degree Celsius, a heart rate of 105 beats per minute (BPM), a blood pressure of 163/83 mmHg, and an oxygen saturation of 94% on 3 liters of oxygen via a nasal cannula. Laboratory evaluation was significant for an elevated WBC count of 22.1, lactic acid of 4.2 millimoles per liter (mmol/L), C-reactive protein (CRP) of 40.6 milligrams per liter (mg/L), uric acid of 9.0 mmol/L, sodium (Na) of 127 mmol/L, and creatinine of 2.7 milligrams per deciliter (mg/dL). On arrival, two sets of blood cultures were collected and grew *Staphylococcus aureus* with non-penicillin-binding protein and resistance to clindamycin and erythromycin just like the synovial fluid joint aspiration from the right second digit. This confirmed septic arthritis with secondary bacteremia leading to septic shock. X-ray of the right second digit was notable for marked soft tissue thickening with underlying bony destruction at the second digit, which may be due to damage from the tophi over time, versus a secondary septic arthritis and osteomyelitis at the distal interphalangeal (DIP) and the proximal interphalangeal (PIP) joints as seen in Figure 1 and in Figure 2. 

Given the aforementioned laboratory results, cultures, and diagnostic imaging, the patient met criteria for sepsis and was treated for septic shock in the setting of presumed osteomyelitis from gout tophi. An orthopedic surgeon was consulted and recommended amputation of the right second digit due to the severity and extent of the joint and bony destruction. Concomitantly, an infectious disease doctor was consulted and recommended 2 grams of intravenous (IV) ceftriaxone antibiotic administration daily, for six weeks. On the second day of admission and after starting antibiotics, it was also recommended to repeat blood cultures to determine the state of bacteremia and perform an echocardiogram during her inpatient stay to rule out bacterial endocarditis. However, the patient's son was her healthcare power of attorney (HPOA) and decided to forgo curative treatment including antibiotic administration, as well as any other inpatient imaging and work-up or plans for outpatient follow-up. Instead, the HPOA expressed wishes for a hospice consult. The patient was discharged on her third day of admission and was sent home with hospice. 

## Discussion

Tophaceous deposits may cause joint destruction, gout nephropathy, and spinal compression and can lead to secondary infections. Our case presents an elderly patient with gout tophi that became an infected ulceration and led to septic arthritis and osteomyelitis and septic shock. Through literature review, there are only a few cases reporting severe infections occurring in the setting of gout tophi. In one case report, a 57-year-old man with a history of tophaceous gout and alcohol abuse presented for severe pain and swelling of the right knee and right ankle for two weeks and was found to have septic arthritis. He was febrile, and his work-up was significant for neutrophilic leukocytosis (16,700 cells/μL), profound hyperuricemia (12.3 mg/dL), and a raised CRP (193 mg/L, normal range < 5). Aspiration of the right knee revealed purulent synovial fluid with 112,000 cells/μL. A Gram stain was positive for Gram-positive cocci, and synovial fluid culture revealed *Streptococcus agalactiae*. Magnetic resonance imaging (MRI) of the knee showed intra-articular fluid collection and edema of adjacent muscles, but it was without evidence of osteomyelitis. In contrast to our patient, orthopedic examination consulted against surgical consultations and the patient's blood cultures were sterile. The patient received empiric treatment of IV daptomycin and levofloxacin for septic arthritis, which was then tailored to penicillin and levofloxacin after the cultures resulted [2].

Another case report presents a 56-year-old male patient who had a total hip replacement for painful osteoarthritis but had no history of gout. His course of hospitalization was complicated by *S. aureus* septicemia caused by an infected jugular vein catheter five days after arthroplasty, and he was treated with IV vancomycin. On the eighth postoperative day, he also developed progressive right wrist pain and fever. Aspiration of the wrist was performed, a white fluid was collected, and the culture from the aspiration was positive for *S. aureus* which confirmed septic arthritis. An emergency dorsal arthrotomy was performed, and white chalky deposits in the wrist were obtained. Additionally, there were severe erosions of the bones of the first carpal row. Pathological examination confirmed the diagnosis of deposits of aggregated crystals of MSU and therefore suggested gout [3]. This case report depicts secondary septic arthritis in the setting of gout similar to the case we present. Moreover, it refers to other reports of tophi existing in the vicinity of necrotic tissues.

Management of concomitant septic arthritis and gout may vary and employ different procedures. For instance, one case study presents a 54-year-old patient with increasing pain in the left knee and unable to bear weight for three days. The patient had a history of gout diagnosed at 17 years of age, exacerbated with high alcohol consumption, and treated with long-term prednisolone and allopurinol. Aspirations of the left knee were described as thick, gritty, and purulent. Cultures grew *Enterococcus faecalis* and *Streptococcus viridans*, suggesting the diagnosis of left knee joint septic arthritis in the setting of tophaceous gout. Initially, the patient was managed with IV vancomycin and flucloxacillin as well as an emergent arthroscopic washout of the joint which drained chalky, gritty accumulations of MSU crystals. Unfortunately, there was minimal clinical improvement, and the patient developed overt sepsis. Therefore, the patient underwent six additional operative debridement and washouts and an extended course of broad-spectrum IV antibiotics, specifically piperacillin with tazobactam and vancomycin. There was still a lack of sepsis control, so the patient had a topical negative pressure wound therapy (NPWT) system which allowed regulated closed infusion of the antibiotic and soaking with an antiseptic solution. Four weeks after commencing the topical negative pressure device, the patient clinically improved and was stable for discharge [4].

In a research study using a hospital database to collect 30 cases of concomitant septic arthritis and gout, treatment options were examined. It was found that two cases employed arthrodesis, 11 cases included debridement, and one case led to an amputation. The remainder of the 30 cases did not include surgical involvement [5]. Typically, antibiotic choice of treatment is guided by cultures and sensitivities. Similarly, surgical interventions and procedures are evaluated on a case-by-case instance. For example, in this research study, patients with severe joint infections required arthrodesis, while the patient with uncontrolled necrotizing fasciitis and septic arthritis required amputation [5]. In our patient case, orthopedic surgery recommended amputation due to the severity and extent of joint and bone destruction of the right second digit.

## Conclusions

Septic arthritis in patients with underlying gouty arthritis can present a clinical diagnostic dilemma, especially when determining whether a patient has a superficial infection at the site of ulceration or a deeper infection involving septic arthritis/osteomyelitis when bacteria is isolated in the area. In elderly patients, altered mental status and septic shock require careful evaluation for sources of infection including from common urinary and respiratory etiologies. However, in the setting of chronic, extensive tophaceous gout with ulcerations and drainage, secondary septic arthritis and osteomyelitis must be considered as the culprit leading to sepsis and septic shock. Management includes broad-spectrum IV antibiotics and oftentimes an orthopedic consultation for debridement of the joint, arthrodesis, or even recommendations for amputation if the joint and bone destruction is severe and extensive, such as the case we present.
